# Editorial: Microbial strategies for drought stress mitigation

**DOI:** 10.3389/fpls.2026.1915379

**Published:** 2026-07-06

**Authors:** Ricardo Aroca, Sofia I. A. Pereira

**Affiliations:** 1 Department of Soil and Plant Microbiology, Estación Experimental del Zaidín (CSIC), Granada, Spain; 2 Universidade Católica Portuguesa, CBQF - Centro de Biotecnologia e Química Fina – Laboratório Associado, Escola Superior de Biotecnologia, Porto, Portugal

**Keywords:** arbuscular mycorrhizal fungi, biofertilizers, biostimulants, metabolomics, plant–microbiome interactions, rhizosphere microbiome, transcriptomics

The contributions gathered in this Research Topic have significantly advanced our understanding of the molecular and physiological mechanisms underlying plant interactions with beneficial microorganisms, particularly their role in enhancing plant performance under drought stress. The first section comprises studies addressing the fundamental mechanisms of arbuscular mycorrhizal fungi (AMF) symbiosis, along with ecological investigations of these interactions. The second section mainly includes field-based studies evaluating the effects of unicellular algae and mulching practices on plant responses to drought conditions.

The studies of Barbosa et al. and Virág et al. provide complementary evidence that the benefits of AMF under drought conditions are strongly dependent on the host genotype and cannot be explained solely by the presence of mycorrhizal associations. Barbosa et al. showed that drought-tolerant common bean genotypes were better able to maintain or increase stable, diverse, and functionally specialized AMF communities under water deficit, whereas drought-sensitive genotypes experienced reduced diversity, loss of specialist taxa, and substantial shifts in mycorrhizal community composition. Similarly, Virág et al. showed that maize genotypes differed markedly in their transcriptional and microbiome responses to *Funneliformis mosseae* colonization, with drought-tolerant plants displaying moderated stress responses and sustained metabolic activity, while drought-sensitive plants underwent extensive transcriptional reprogramming, persistent stress signaling and compensatory metabolic activation. Together, these studies indicate that successful drought adaptation depends on the capacity of plants to establish and maintain functionally effective interactions with AMF under stress. Moreover, the two studies address complementary yet interconnected levels of biological organization in plant-AMF interactions under drought stress. Barbosa et al. focused on the ecological dimension of these interactions, demonstrating that drought tolerance is associated with the ability of host plants to maintain stable and functionally specialized AMF communities. Notably, greater AMF community stability was linked to enhanced root biomass, highlighting the close relationship between microbiome organization and plant performance. Virág et al. explored the molecular basis of these interactions through integrated transcriptomic and metatranscriptomic analyses, revealing how AMF modulate host regulatory networks and shape the functional activity of the root-associated microbiome. Together, these studies provide a multiscale perspective of drought adaptation, linking microbiome structure and stability with the molecular mechanisms that underpin plant resilience under water deficit. Future agricultural strategies should move beyond the selection of efficient microbial inoculants alone and incorporate host traits that govern microbiome recruitment and functionality.

On the other hand, Moncada et al. moved beyond conventional evaluations of microbial consortia based on plant growth and nutrient acquisition by employing untargeted LC-MS metabolomics to elucidate the biochemical mechanisms that underlying microbial-mediated drought tolerance. Their results revealed that the efficacy of microbial biostimulants is highly consortium-specific, with different microbial combinations triggering distinct metabolic responses in the host plant. Notably, the most effective consortium, composed of an AMF, a yeast, and a plant growth-promoting bacterium (PGPB), promoted the accumulation of flavonoids, phenolic compounds, fatty acid derivatives, and hormone-related metabolites associated with stress protection. By linking microbial inoculation to specific metabolic signatures, the study provided valuable mechanistic insights into how beneficial microorganisms enhance plant resilience under water deficit. The findings demonstrated that microbial-mediated drought tolerance depends not only on improved nutrient acquisition but also on the capacity of specific microbial consortia to reprogram plant metabolism and activate protective biochemical pathways.

Other microorganisms that have been less extensively studied as biostimulants are unicellular algae. In this regard, Molina-Favero et al. found that the unicellular alga *Scenedesmus obliquus* promoted root colonization by the PGPB *Azospirillum argentinensis*. Moreover, under field conditions relying exclusively on natural rainfall, the highest maize grain yield was observed following co-inoculation with the algae and two PGPB strains under conventional tillage management. These plants also exhibited the highest water-use efficiency. Therefore, the microbial consortium evaluated in this study may represent a promising tool for improving maize production under rainfed conditions.

Besides the direct inoculation of microorganisms, agricultural practices can also modify the rhizosphere microbiome, thereby enhancing crop drought tolerance. In this regard, Wu et al. found that plastic film mulching, particularly film-side planting, increased maize yield. Interestingly, however, this practice reduced the abundance of mycorrhiza-associated fungi and nitrifying microorganisms while favoring anaerobic microbial communities. Therefore, although mulching may be beneficial in the short term, its long-term effects remain uncertain due to the decline in these beneficial microorganisms.

Based on the studies published in this Research Topic, it can be concluded that the effects of beneficial microorganisms on plant performance under drought conditions are influenced by multiple factors, including host-specific interactions, agricultural practices, and the composition of the microbial consortium. Consequently, further fundamental and applied research is required to elucidate how these factors shape the outcomes of plant–microbe interactions across diverse environmental conditions.

